# A Case of Implant Failure in Partial Wrist Fusion Applying Magnesium-Based Headless Bone Screws

**DOI:** 10.1155/2016/7049130

**Published:** 2016-10-05

**Authors:** Alice Wichelhaus, Judith Emmerich, Thomas Mittlmeier

**Affiliations:** Department of Trauma, Hand and Reconstructive Surgery, University Faculty of Medicine Rostock, Schillingallee 35, 18057 Rostock, Germany

## Abstract

This article presents a case of implant failure resulting in mechanical instability of a scaphotrapezotrapezoideal arthrodesis using magnesium-based headless bone screws. During revision surgery osteolysis surrounding the screws was observed as well as degraded screw threads already in existence at 6 weeks after implantation. The supposed osseous integration attributed to magnesium-based screws could not be reproduced in this particular case. Thus, it can be reasoned that the use of magnesium-based screws for partial wrist arthrodesis cannot be encouraged, at least not in dual use.

## 1. Introduction

Triscaphoid arthrodesis is an established treatment option for symptomatic osteoarthritis of the scaphotrapezotrapezoideal joint [1, 2]. Long-term effects of the hereby diverted force transmission can be arthritic changes of the adjacent carpal joints [2, 3], so that the younger the patient is, the more probable the need for revision surgery is. The use of biodegradable implants has the basic advantage to render implant removal unnecessary. In the last decade, biodegradable magnesium-based implants have been tested in clinical trials with promising results [4, 5]. A case of a severe adverse event using magnesium-based screws for partial wrist fusion is reported.

## 2. Case Presentation

The patient is a 42-year-old, right-hand dominant manual worker who presented himself after a fall on the extended right hand. Previous trauma could not be recalled. Preexisting medical conditions, smoking, long-term medication, allergic diathesis, or the like were negated.

**Figure 1:**
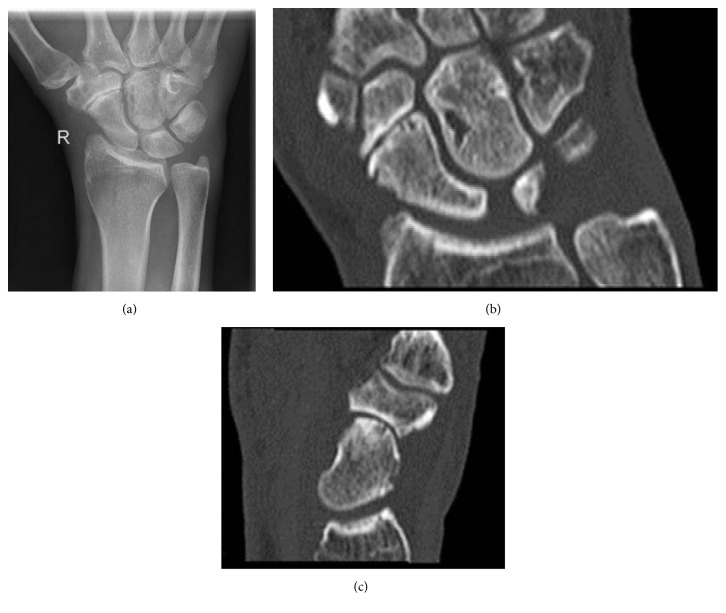
(a) X-ray of right wrist showing fracture of the scaphoid and preexistent osteoarthritis of the STT-joint. (b, c) Preoperative CT-scan demonstrating fracture of scaphoid classified as Herbert B2 but also clearly demonstrating degenerative changes of the carpal bones.

At initial presentation a subtle swelling of the right wrist was stated as well as tenderness on palpation in the scaphoid region, especially the snuff box. Peripheral sensorimotor deficits were not detected. Radiographic imaging revealed a scaphoid fracture, classified as B2 according to the Herbert-classification [6]. Both the native radiographs and the computed tomography showed a preexistent scaphotrapezotrapezoidal (STT) arthritis and the configuration of static dorsally intercalated segmental instability (DISI) (Figures 1(a)–1(c)).

**Figure 2:**
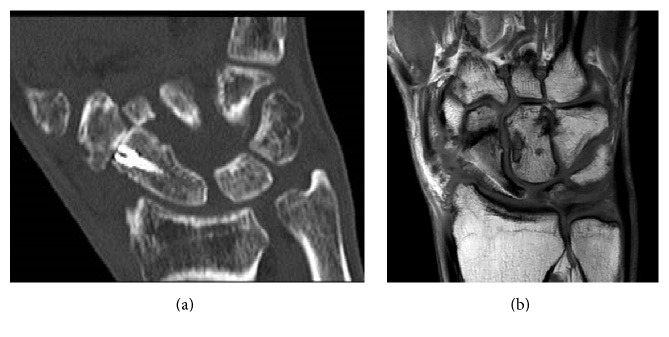
(a) CT-scan showing consolidated fracture of the scaphoid and progression of osteoarthritis in STT-joint. (b) MRI-scan showing healed fracture of the scaphoid with regular perfusion.

The patient insisted on having been completely free of pain and practicing undisturbed skilled manual work prior to the accident. Therefore he gave his informed consent to only address the scaphoid fracture operatively although it was well explained that persistent discomfort was probable due to the preexisting osteoarthritis. Fracture reduction was achieved via palmar approach and retained with a cannulated headless bone screw (KLS Martin, Tuttlingen, Germany). Postoperatively a wrist-cast manumitting the metacarpophalangeal joint was applied for 6 weeks. After fracture consolidation was ensured by CT-scan (Figure 2(a)), the patient still suffered from significant pain on load bearing of the affected wrist and, respectively, the base of the thumb. Plain radiographs visualized progression of the preexistent osteoarthritis of the STT-joint.

MRI-scans excluded circulatory disorders of the scaphoid (Figure 2(b)).

**Figure 3:**
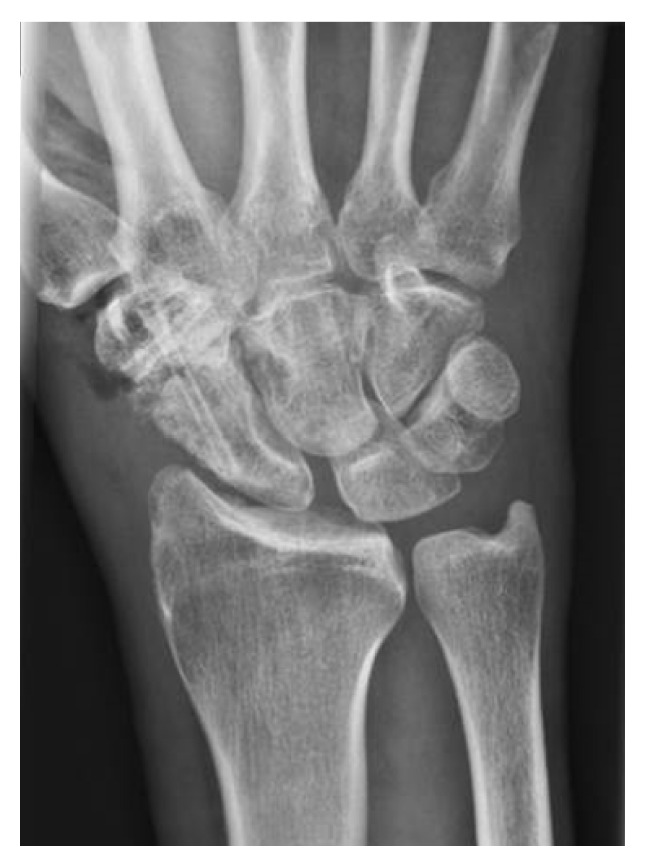
X-ray right wrist a.p. following STT-fusion with magnesium-based screws.

Despite functional training and physiotherapy, wrist function remained restricted and painful. To minimize the handicap and restore the ability to work, arthrodesis of the STT-joint was performed in February 2014 (Figure 3). As further revision surgery is not unlikely after partial wrist fusion, two biodegradable magnesium-based headless bone screws (Magnezix®, Syntellix, Hannover, Germany) were used along with autologous bone grafting from the iliac crest.

**Figure 4:**
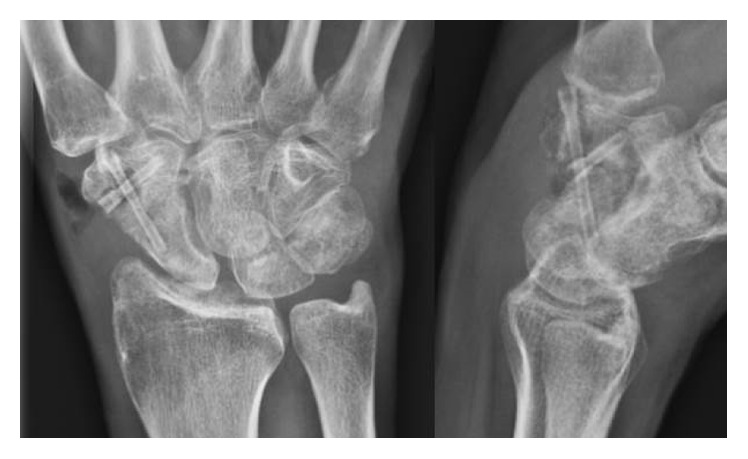
X-ray of the same wrist as in Figure 3, 6 weeks after arthrodesis showing loosening and backing out of both screws.

Magnesium-based implants have been successfully applied by the authors for corrective surgery of the forefoot including revision surgery and in two cases for reconstruction of comminuted distal humerus fractures. After the operation cast immobilization was maintained for 6 weeks. Several days after removal of the plaster cast, the patient complained about painful paraesthesia in the dorsal thumb region. He developed severe swelling of the radial and dorsal aspect of the wrist. X-rays of the wrist in two planes revealed loosening and backing out of the screws causing irritation to the dorsal ramus of the superficial radial nerve (Figure 4).

**Figure 5:**
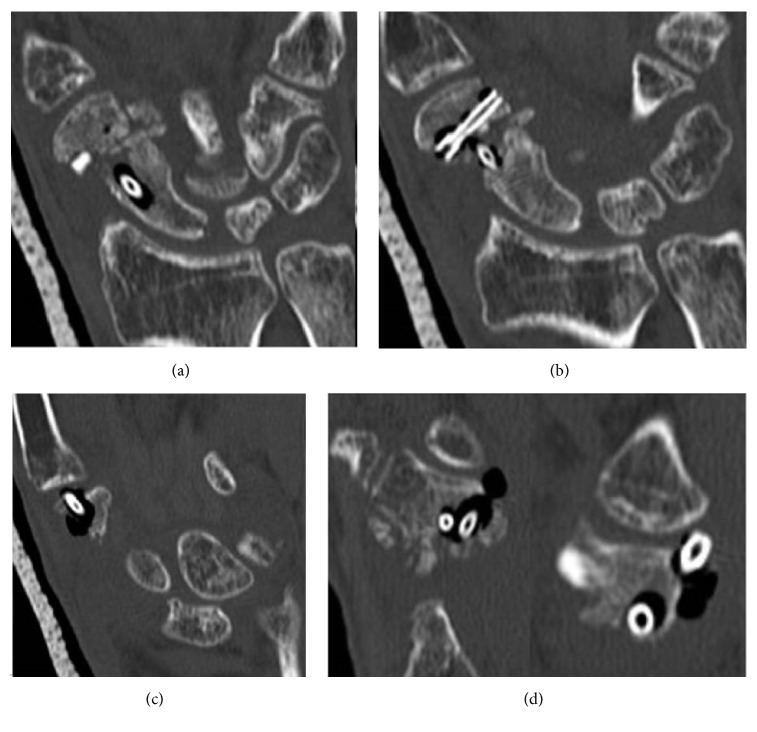
CT-scan taken 6 weeks after triscaphoid arthrodesis.

CT-scans showed osteolytic seams around both screws with cystic formations in the scaphoid, trapezium, and trapezoid (Figures 5(a)–5(d)).

Subcutaneous gas accumulations could not be palpated. During revision surgery large voids around the inserted screws were found. The two screws could be removed with tweezers without using a drill. The screw threads were barely recognizable; the tissue surrounding the screws was discolored blackish. The surrounding soft tissue showed severe synovitis with anthracite pigmentation. Unfortunately, no tissue was retained for histological assessment. As the arthrodesis was not consolidated by any means, rearthrodesis was necessary using Kirschner-wires.

In autumn 2014 progressive DISI-deformity and symptomatic periscaphoid osteoarthritis called for midcarpal arthrodesis with extirpation of the scaphoid as a salvage procedure. During the course of this operation, synovia was sampled for histological examination. Microscopy revealed signs of chronical hyperplastic synovitis with a wide range of proliferated synoviocytes throughout the whole sample. Hemosiderin pigment in macrophages was detected as well as multinucleated giant cells equaling foreign body reaction. Extraneous material could not be visualized under plane polarising light. Pigment due to metallic abrasive wear was not found any more than signs for pigmented villonodular synovitis.

**Figure 6:**
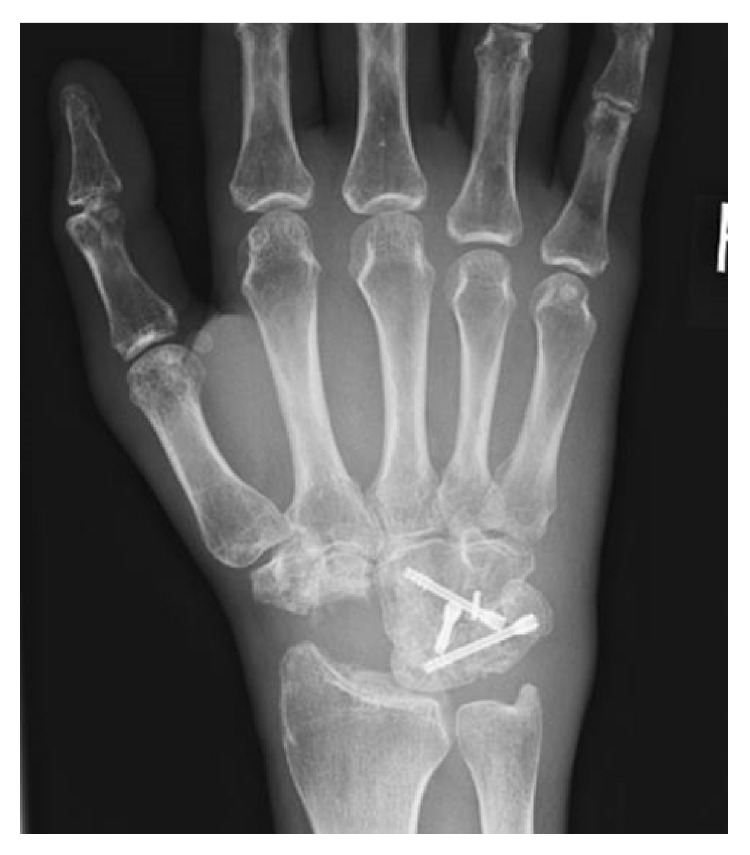
X-ray right wrist a.p. showing consolidated 4-corner fusion.

The 4-corner fusion consolidated in time (Figure 6).

The patient has not been free of wrist pain ever since the first operation. Wrist range of motion remained restricted with ROM extension/flexion of 30–0–15° and ulnar/radial deviation of 15–0–10°. Grip strength measured with a Jamar-dynamometer achieved 1/3 of the uninjured contralateral side. So far, the patient did not settle for total wrist arthrodesis considering the hitherto conflicted devolution of disease. Till today resumption of manual work has not been possible.

## 3. Discussion

STT-fusions are performed for degenerative arthritic changes of the STT-joint, scaphoid instability, and advanced necrosis of the lunate [3, 7]. Triscaphoid arthrodesis has been proposed to redirect force transmission away from the lunate in way of stabilizing the radial column. The altered joint surfaces are uncoupled. Different stabilization techniques have been described using K-wires, headless bone screws, or anatomically shaped angular stable plates. Regardless of the technique, good results in terms of function and pain reduction are reported accompanied by a loss of grip strength of 20–40%. Nonunion is reported in up to 15% of the cases irrespective of the used material [3, 7]. For carpal arthrodesis a consolidation period of 8–12 weeks is assumed. In the reported case, the observable migration of the screws was caused by loss of retention force due to heavy corrosion of the screw threads present six weeks after implantation of the screws. Based on the published data on the magnesium-based screws, a slower degradation of the bolts had to be expected [8–10]. In animal studies, good biocompatibility has been described [8–11] for the magnesium-based screws. The degradation process of the magnesium material is said to have no adverse effect on bone healing but rather to promote bone formation at the screw-bone interface [8–11]. The mechanical strength of magnesium screws was tested to be equal to titanium-based screws [5]. A higher mechanical strength was determined for magnesium-based screws compared to other biodegradable polymer-based interference screws [12]. Reviewing the literature, reports of allergic reactions to magnesium-containing implants could not be found. Quite the contrary, magnesium-based implants attested little allergenic potential [13, 14]. Some authors even suggest to resign from using conventional metal alloys in patients with known allergic diathesis since their corrosion products showed cytotoxic and proinflammatory effects. In contrast the magnesium-based substitutes are said to deliver ions during their degradation that are needed for the physiological metabolism [14].

Despite the reported good osseointegration properties of magnesium screws, severe osteolysis surrounding the implants occurred in the present case. In a clinical application study good results were reported using magnesium-based headless bone screws for Chevron osteotomy [5]. However these good results could not be reproduced in the partial wrist fusion in this case. After revision surgery using titanium-based implants, solid osseous consolidation of the fusion could be achieved. Since headless bone screws were used in both operations, it can be assumed that the initial treatment failure is not due to the nature of the screws but to their metallic composition. Our observations are supported by an animal study which also stated poor osseointegration when using magnesium-based screws resulting in mechanical instability [15].

## 4. Conclusion

Early degradation of the screws leads to mechanical instability resulting in nonunion and osteolysis of the three carpal bones. Due to this disappointing result of the operation with premature mechanical instability, we cannot support the use of magnesium-based screws for partial wrist arthrodesis, at least not in dual use.

## References

[1] Goubier J.-N., Bauer B., Alnot J.-Y., Teboul F. (2006). Scapho-trapezio-trapezoidal arthrodesis for scapho-trapezio-trapezoidal osteoarthritis. *Chirurgie de la Main*.

[2] Kalb K., Fuchs V., Bartelmann U., Schmitt R., Landsleitner B. (2001). Experiences with scapho-trapezio-trapezoid arthrodesis. A retrospective analysis. *Handchirurgie Mikrochirurgie Plastische Chirurgie*.

[3] Meier R., Busche M., Krettek C. (2005). Force distribution in the wrist following scaphotrapeziotrapezoid arthrodesis. *Der Unfallchirurg*.

[4] Farraro K. F., Kim K. E., Woo S. L.-Y., Flowers J. R., McCullough M. B. (2014). Revolutionizing orthopaedic biomaterials: the potential of biodegradable and bioresorbable magnesium-based materials for functional tissue engineering. *Journal of Biomechanics*.

[5] Windhagen H., Radtke K., Weizbauer A. (2013). Biodegradable magnesium-based screw clinically equivalent to titanium screw in hallux valgus surgery: short term results of the first prospective, randomized, controlled clinical pilot study. *BioMedical Engineering OnLine*.

[6] Amadio P. C., Moran S. L., Green D. P. (2005). Fractures of the carpal bones. *Operative Hand Surgery*.

[7] Meier R., Prommersberger K. J., Krimmer H. (2003). Scapho-trapezio-trapezoid arthrodesis (triscaphe arthrodesis). *Handchirurgie, Mikrochirurgie, Plastische Chirurgie*.

[8] Chaya A., Yoshizawa S., Verdelis K. (2015). In vivo study of magnesium plate and screw degradation and bone fracture healing. *Acta Biomaterialia*.

[9] Diekmann J., Bauer S., Weizbauer A. (2016). Examination of a biodegradable magnesium screw for the reconstruction of the anterior cruciate ligament: a pilot in vivo study in rabbits. *Materials Science and Engineering C*.

[10] Waizy H., Diekmann J., Weizbauer A. (2014). In vivo study of a biodegradable orthopedic screw (MgYREZr-alloy) in a rabbit model for up to 12 months. *Journal of Biomaterials Applications*.

[11] Han P., Cheng P., Zhang S. (2015). In vitro and in vivo studies on the degradation of high-purity Mg (99.99wt%) screw with femoral intracondylar fractured rabbit model. *Biomaterials*.

[12] Ezechieli M., Ettinger M., König C. (2014). Biomechanical characteristics of bioabsorbable magnesium-based (MgYREZr-alloy) interference screws with different threads. *Knee Surgery, Sports Traumatology, Arthroscopy*.

[13] Witte F., Abeln I., Switzer E., Kaese V., Meyer-Lindenberg A., Windhagen H. (2008). Evaluation of the skin sensitizing potential of biodegradable magnesium alloys. *Journal of Biomedical Materials Research Part A*.

[14] Witte F., Calliess T., Windhagen H. (2008). Biodegradable synthetic implant materials: clinical applications and immunological aspects. *Der Orthopäde*.

[15] Thormann U., Alt V., Heimann L. (2015). The biocompatibility of degradable magnesium interference screws: an experimental study with sheep. *BioMed Research International*.

